# Supplementary material for: Profound Impact of Local Climatic Conditions on IgE Sensitization Profiles: Evidence from Argentine Cities
Source: Int J Mol Sci. 2025 Dec 16;26(24):12101. doi: 10.3390/ijms262412101 (PMC12733070; doi:10.3390/ijms262412101)
Supplement: Supplementary file 1 [file ijms-26-12101-s001.zip › Table S1.pdf]

Patient	Sex	Age	City	Symptoms		
**1**	36	f	Bahia Blanca	RC		D
**2**	21	f	Bahia Blanca	RC	AS	
**3**	56	m	Bahia Blanca	RC	AS	D
**4**	31	m	Bahia Blanca			D
**5**	28	m	Bahia Blanca	RC		D
**6**	61	m	Bahia Blanca	RC		D
**7**	33	m	Bahia Blanca	RC		D
**8**	28	m	Bahia Blanca	RC		
**9**	27	f	Bahia Blanca	RC	AS	
**10**	18	f	Bahia Blanca	RC		
**11**	28	f	Bahia Blanca	RC	AS	
**12**	19	m	Bahia Blanca	RC		D
**13**	49	f	Bahia Blanca	RC		
**14**	24	m	Bahia Blanca	RC		
**15**	24	m	Bahia Blanca			D
**16**	26	m	Bahia Blanca	RC		
**17**	28	f	Bahia Blanca	RC		
**18**	25	f	Bahia Blanca	RC		
**19**	55	f	Bahia Blanca	RC		D
**20**	41	f	Bahia Blanca	RC		
**21**	33	m	Bahia Blanca	RC		
**22**	45	f	Bahia Blanca	RC		
**23**	32	m	Bahia Blanca	RC		D
**24**	29	m	Bahia Blanca	RC	AS	D
**25**	50	f	Bahia Blanca	RC		D
**26**	45	m	Bahia Blanca	RC		D
**27**	20	f	Bahia Blanca	RC		
**28**	45	m	Bahia Blanca	RC		
**29**	48	f	Bahia Blanca	RC		D
**30**	44	m	Bahia Blanca	RC		D
**31**	28	m	Bahia Blanca	RC		D
**32**	27	f	Bahia Blanca	RC	AS	
**33**	18	m	Bahia Blanca	RC		
**34**	21	f	Bahia Blanca	RC	AS	
**35**	46	m	Bahia Blanca	RC		
**36**	45	m	Bahia Blanca	RC		
**37**	50	f	Bahia Blanca	RC		
**38**	38	f	Bahia Blanca	RC		D
**39**	36	m	Bahia Blanca	RC		D
**40**	23	f	Bahia Blanca			D
**41**	24	m	Bahia Blanca	RC		
**42**	24	m	Bahia Blanca	RC		D
**43**	49	f	Bahia Blanca	RC	AS	D
**44**	32	f	Bahia Blanca	RC		D
**45**	47	m	Bahia Blanca	RC		
**46**	36	m	Bahia Blanca	RC	AS	
**47**	47	m	Bahia Blanca	RC		D
**48**	19	m	Bahia Blanca	RC	AS	D
**49**	47	m	Bahia Blanca			D
**50**	30	m	Bahia Blanca	RC	AS	D
**51**	26	f	Bahia Blanca	RC		
**52**	19	m	Bahia Blanca	RC	AS	D
**53**	46	m	Bahia Blanca	RC	AS	D
**54**	49	f	Bahia Blanca	RC	AS	
**55**	19	f	Bahia Blanca	RC		D
**56**	36	f	Bahia Blanca	RC		
**57**	23	f	Bahia Blanca	RC		
**58**	33	f	Bahia Blanca	RC	AS	
**59**	52	f	Bahia Blanca	RC		
**60**	25	m	Bahia Blanca	RC	AS	
**61**	38	f	Bahia Blanca	RC		D
**62**	30	f	Bahia Blanca	RC	AS	D
**63**	25	m	Bahia Blanca	RC		
**64**	29	m	Bahia Blanca	RC	AS	D
**65**	35	m	Bahia Blanca	RC		
**66**	22	f	Bahia Blanca	RC		
**67**	22	m	Bahia Blanca	RC		
**68**	29	m	Bahia Blanca	RC		
**69**	25	f	Bahia Blanca	RC		
**70**	18	f	Bahia Blanca	RC		
**71**	37	m	Bahia Blanca	RC		D
**72**	45	m	Bahia Blanca	RC	AS	D
**73**	47	f	Bahia Blanca	RC	AS	D
**74**	30	m	Bahia Blanca	RC		
**75**	48	f	Bahia Blanca	RC		D
**76**	49	m	Bahia Blanca	RC		D
**77**	33	m	Bahia Blanca	RC		D
**78**	23	m	Bahia Blanca	RC		D
**79**	28	f	Bahia Blanca	RC		D
**80**	43	m	Bahia Blanca	RC		D
**81**	35	m	Bahia Blanca	RC		
**82**	46	f	Bahia Blanca	RC		
**83**	24	m	Bahia Blanca	RC		D
**84**	32	f	Bahia Blanca	RC		D
**85**	24	m	Bahia Blanca	RC		D
**86**	40	m	Bahia Blanca	RC		
**87**	42	f	Bahia Blanca	RC	AS	
**88**	34	m	Bahia Blanca	RC		D
**89**	47	m	Bahia Blanca	RC	AS	D
**90**	46	m	Bahia Blanca	RC		D
**91**	38	f	Bahia Blanca	RC	AS	D
**92**	18	m	Bahia Blanca	RC	AS	D
**93**	42	m	Bahia Blanca	RC		D
**94**	35	m	Bahia Blanca	RC		D
**95**	22	f	Bahia Blanca			D
**96**	31	m	Bahia Blanca	RC		
**97**	40	m	Bahia Blanca	RC		D
**98**	20	f	Bahia Blanca	RC		
**99**	50	m	Bahia Blanca	RC		
**100**	20	f	Bahia Blanca			D
**101**	58	f	La Plata			
**102**	49	m	La Plata	RC	AS	D
**103**	24	f	La Plata	RC		D
**104**	28	f	La Plata			D
**105**	16	f	La Plata	RC		
**106**	50	f	La Plata	RC	AS	
**107**	39	f	La Plata	RC	AS	D
**108**	42	m	La Plata	RC		
**109**	52	f	La Plata	RC	AS	
**110**	22	m	La Plata	RC		
**111**	50	f	La Plata	RC	AS	
**112**	17	f	La Plata	RC		D
**113**	32	f	La Plata	RC	AS	
**114**	26	f	La Plata	RC	AS	
**115**	46	m	La Plata	RC	AS	
**116**	22	f	La Plata	RC		
**117**	42	f	La Plata	RC	AS	
**118**	62	f	La Plata	RC	AS	
**119**	25	m	La Plata	RC		
**120**	29	f	La Plata	RC	AS	
**121**	23	f	La Plata	RC		D
**122**	29	f	La Plata	RC	AS	
**123**	27	m	La Plata	RC	AS	
**124**	39	f	La Plata	RC	AS	
**125**	21	f	La Plata	RC		
**126**	25	f	La Plata	RC		
**127**	36	f	La Plata	RC		
**128**	39	f	La Plata	RC	AS	D
**129**	33	f	La Plata	RC	AS	D
**130**	39	f	La Plata	RC		D
**131**	58	m	La Plata	RC		
**132**	30	m	La Plata			D
**133**	20	f	La Plata	RC	AS	
**134**	15	f	La Plata	RC	AS	
**135**	25	f	La Plata	RC	AS	
**136**	18	m	La Plata	RC		D
**137**	24	m	La Plata	RC		
**138**	38	f	La Plata	RC	AS	D
**139**	52	m	La Plata	RC	AS	
**140**	28	f	La Plata	RC		
**141**	45	f	La Plata	RC		
**142**	43	f	La Plata	RC		
**143**	39	f	La Plata	RC		
**144**	29	f	La Plata			D
**145**	26	m	La Plata	RC		
**146**	58	f	La Plata	RC		D
**147**	47	f	La Plata	RC		
**148**	37	m	La Plata	RC		D
**149**	25	f	La Plata			D
**150**	26	f	La Plata	RC		
**151**	25	f	La Plata	RC		
**152**	42	f	La Plata	RC		
**153**	26	m	La Plata	RC		
**154**	26	f	La Plata	RC		
**155**	22	m	La Plata	RC		D
