# Supplementary material for: Profound Impact of Local Climatic Conditions on IgE Sensitization Profiles: Evidence from Argentine Cities
Source: Int J Mol Sci. 2025 Dec 16;26(24):12101. doi: 10.3390/ijms262412101 (PMC12733070; doi:10.3390/ijms262412101)
Supplement: Supplementary file 1 [file ijms-26-12101-s001.zip › Table S2.pdf]

Allergen source		Allergen	UniProt Accession n.	Rec. or natural	Source	Route of exposure	Function of the protein/Allergen family
Tree pollen	*Birch*	Bet v 1	P15494	R	In-house	r	PR-10
		Bet v 2	P25816.1	R	In-house	r	Profilin
		Bet v 4	Q39419	R	In-house	r	Polcalcin
	Olive	Ole e 1	P19963	R	In-house	r	Ole e 1-related protein
		Ole e 2	O24169	R	In-house	r	Profilin
		Ole e 3	O81092	R	In-house	r	Polcalcin
		Ole e 5	Q8L5E0	R	In-house	r	Superoxide dismutase
		Ole e 6	O24172	R	In-house	r	Oleaceae group 6
		Ole e 8	Q9M7R0	R	In-house	r	Calcium-binding protein (Polcalcin)
		Ole e 10	Q84V39	R	In-house	r	Glycosyl hydrolase
	Plane tree	Pla a 1	Q8GT41	R	In-house	r	Invertase inhibitor
Grass pollen	*Timothy grass*	Phl p 1		R	Commercial/Biomay	r	Grass group 1 (Beta-Expansin)
		Phl p 2	P43214	R	In-house	r	Grass group 2/3
		Phl p 3	Q69B42	R	In-house	r	Grass gorup 3
		Phl p 5b	Q40960	R	In-house	r	Grass group 5
		Phl p 6	O65868	R	In-house	r	Grass group 5/6
		Phl p 7	O82040	R	In-house	r	Polcalcin
		Phl p 12	P35079	R	In-house	r	Profilin
Weed pollen	*Ragweed*	Amb a 1	P27759	R	In-house	r	Pectate lyase
		Amb a 4	D4IHC0	R	In-house	r	Defensin-like protein
		Amb a 8	Q2KN24	R	In-house	r	Profilin
	Mugwort	Art v 1	Q84ZX5	R	In-house	r	Defensin-like protein
		Art v 6	AOPJ16	R	In-house	r	Pectate lyase
	Plantain	Pla l 1	Q8GT41	R	In-house	r	Ole e 1-related protein
	Wall pellitory	Par j 2	P55958	R	In-house	r	nsLTP, type 1
Mould	*Alternaria*	Alt a 1	Q6Q128	R	In-house	r	Acidic glycoprotein
	Aspergillus	Asp f 1	P67875	R	In-house	r	Mitogillin family (Ribonuclease)
		Asp f 3	O43099	R	In-house	r	Peroxysomal protein
		Asp f 6	Q92450	R	In-house	r	Mn superoxide dismutase
	Cladosporium	Cla h 8		R	Commercial/Biomay	r	Mannitol dehydrogenase
Mite & Cockroach	*House dust mite*	Der p 1		N	Commercial/Citeq	r	Group 1 mite allergen (Cysteine protease)
		Der p 2		N	Commercial/Inbio	r	Group 2 mite allergen (NPC2 family)
		Der p 4	Q9Y197	R	In-house	r	Group 4 mite allergen (Alpha-amylase)
		Der p 5	P14004	R	In-house	r	Group 5/21 mite allergen
		Der p 7	P49273	R	In-house	r	Group 7 mite allergen
		Der p 10	O18416	R	In-house	r	Group 10 mite allergen (Tropomyosin)
		Der p 18	Q4JK71	R	In-house	r	Chitin-binding domain
		Der p 21	Q2L7C5	R	In-house	r	Group 5/21 mite allergen
		Der p 23	L7N6F8	R	In-house	r	Peritrophin-like protein
		Der p 37	A0A3Q0KAE3	R	In-house	r	Chitin-binding domain
	Blomia tropicalis	Blo t 1	Q95PJ4	R	In-house	r	Cysteine protease
		Blo t 2	Q1M2P1	R	In-house	r	ML-domain lipid binding protein
		Blo t 5	O96870	R	In-house	r	Group 5/21 mite allergen
		Blo t 8	C8CGT7	R	In-house	r	Glutathione S-transferase
		Blo t 10	A7XZI4	R	In-house	r	Tropomyosin
		Blo t 12	Q17282	R	In-house	r	Peritrophin-A domain containing protein
		Blo t 13	Q17284	R	In-house	r	Fatty acid-binding protein
		Blo t 21	A7IZE9	R	In-house	r	Unknown
	Cockroach	Bla g 1	Q9UAM5	R	In-house	r	Cockroach group 1
		Bla g 2	P54958	R	In-house	r	Aspartic protease
		Bla g 5	O18598	R	In-house	r	Glutathione-S-transferase
		Bla g 7	Q9NG56	R	In-house	r	Tropomyosin

Allergen-category	Allergen-source	Allergen	Accession number	Rec. or natural	Source	Route of exposure	Function of the protein/Allergen family
Animal dander	*Dog*	Can f 1	O18873	R	In-house	r	Lipocalin
		Can f 2	O18874	R	In-house	r	Lipocalin
		Can f 3		N	Commercial/Sigma	r	Serum albumin
		Can f 4	D7PBH4	R	In-house	r	Lipocalin
	Cat	Fel d 1	P30438; P30440	R	In-house	r	Uteroglobin
		Fel d 2		N	Commercial/Sigma	r	Serum albumin
		Fel d 3	Q8WNR9	R	In-house	r	Cysteine protease inhibitor
		Fel d 4	Q5VFH6	R	In-house	r	Lipocalin
	Mouse	Mus m 1	P02762	R	In-house	r	Lipocalin
	Horse	Equ c 1	Q95182	R	In-house	r	Lipocalin
		Equ c 3		N	Commercial/Sigma	r	Serum albumin
Contact allergens	*Latex*	Hev b 3	O82803	R	In-house	r, s	Small rubber particle protein
		Hev b 5	Q39967	R	In-house	r, s	Acidic protein
		Hev b 8	Q9STB6.1	R	In-house	r, s	Profilin
Plant-derived food	*Apple*	Mal d 1	Q9SYW3	R	In-house	f	PR-10
	Peach	Pru p 1	Q2I6V8	R	In-house	f	PR-10
		Pru p 3	Q9LED1	R	In-house	f, s	nsLTP, type 1
	Peanut	Ara h 1	P43238	R	In-house	f	Storage protein, 7S globulin (Vicilin)
		Ara h 2	Q6PSU2	R	In-house	f	Storage protein, 2S albumin
		Ara h 3	O82580	R	In-house	f	Storage protein, 11S globulin (Legumin)
		Ara h 6	Q647G9	R	In-house	f	Storage protein, 2S albumin
		Ara h 8	B0YIU5	R	In-house	f	PR-10
		Ara h 9	B6CEX8	R	In-house	f	nsLTP, type 1
	Hazelnut	Cor a 1	Q08407	R	In-house	f	PR-10
	Soy	Gly m 4	P26987	R	In-house	f	PR-10
		Gly m 5		N	Commercial/Inbio	f	Storage protein, 7S globulin (Vicilin)
		Gly m 6		N	Commercial/Inbio	f	Storage protein, 11S globulin (Legumin)
	Wheat	Tri a 20	P08453.1	R	In-house	f	Gamma-gliadin
		Tri a 37	Q9T0P1	R	In-house	f	Thionins (Alpha-purothionin)
Animal-derived food	*Cow's milk*	Bos d 4	P00711	R	In-house	f	Alpha-lactalbumin
		Bos d 5		N	Commercial/Sigma	f	Beta-lactoglobulin
		Bos d 8		N	Commercial/Sigma	f	Casein
		Bos d Lactoferrin		N	Commercial/Sigma	f	Transferrin
	Egg	Gal d 1		N	Commercial/Sigma	f	Ovomucoid
		Gal d 2		N	Commercial/Sigma	f	Ovalbumin
		Gal d 3		N	Commercial/Sigma	f	Ovotransferrin (Conalbumin)
		Gal d 4		N	Commercial/Sigma	f, r	Lysozyme C
	Codfish	Gad c 1	P02622	R	In-house	f	Parvalbumin
	Shrimp	Pen m 1	A1KYZ2	R	In-house	f	Tropomyosin
		Pen m 2	Q8I9P7	R	In-house	f	Arginine kinase
	Snail	Hel as 1	O97192	R	In-house	f	Tropomyosin
Insect venom	*Bee*	Api m 1	P00630	R	In-house	sys	Insect venom, Phospholipase A2
		Api m 2	Q08169	R	In-house	sys	Insect venom, Hyaluronidase
	Wasp	Ves v 1	P49369	R	In-house	sys	Insect venom, Phospholipase A1
		Ves v 2	P49370	R	In-house	sys	Hyaluronidase
		Ves v 5	Q05110	R	In-house	sys	Insect venom, Antigen 5
Parasites	*Anisakis*	Ani s 3	Q9NAS5	R	In-house	f	Tropomyosin
CCD-markers	*Horseradish*	HRP		R	Commercial/Sigma	f, r	Bromelain (CCD-Marker)
