# Supplementary material for: Profound Impact of Local Climatic Conditions on IgE Sensitization Profiles: Evidence from Argentine Cities
Source: Int J Mol Sci. 2025 Dec 16;26(24):12101. doi: 10.3390/ijms262412101 (PMC12733070; doi:10.3390/ijms262412101)
Supplement: Supplementary file 1 [file ijms-26-12101-s001.zip › Table S3 and 4.pdf]

Table S3

Patients from La Plata			Frequency		IgE (ISU)			
		Total (n)	n	%	Minimum	Median	Maximum	SD
Respiratory	Der p 23	43	32	74%	0.21	14.02	183.71	50.70
	Der p 2	43	24	56%	0.25	36.94	236.20	53.46
	Der p 1	43	23	53%	0.17	24.77	161.83	44.02
	Der p 7	43	17	40%	0.14	13.82	197.64	54.62
	Der p 21	43	16	37%	0.12	21.27	214.20	66.98
	Der p 5	43	15	35%	0.15	46.93	239.22	69.85
	Fel d 1	43	13	30%	0.52	1.90	71.60	22.81
	Blo t 21	43	12	28%	1.94	8.74	69.34	19.49
	Der p 37	43	10	23%	0.14	4.19	163.44	47.76
	Blo t 5	43	10	23%	0.12	3.59	19.45	7.55
	Phl p 1	43	8	19%	0.33	2.00	59.44	18.96
	Alt a 1	43	8	19%	0.21	10.24	45.69	16.55
	Blo t 2	43	7	16%	0.14	0.23	0.41	0.11
	Der p 4	43	7	16%	0.15	0.17	2.25	0.72
	Equ c 3	43	6	14%	0.12	0.25	1.45	0.51
	Amb a 1	43	6	14%	0.39	0.99	16.74	5.87
	Blo t 13	43	6	14%	0.12	2.56	49.32	17.61
	Amb a 8	43	5	12%	0.23	0.72	5.65	2.04
	Amb a 4	43	5	12%	0.12	0.28	17.29	6.82
	Fel d 3	43	5	12%	0.21	0.41	22.16	8.55
	Fel d 4	43	5	12%	0.14	0.17	0.37	0.08
	Der p 18	43	5	12%	0.12	0.61	19.37	7.60
	Art v 1	43	4	9%	0.15	0.66	12.91	5.39
	Fel d 2	43	4	9%	0.21	0.71	1.63	0.61
	Ole e 2	43	4	9%	0.14	0.97	16.73	6.98
	Phl p 12	43	3	7%	0.58	4.32	28.79	12.51
	Asp f 3	43	3	7%	0.43	0.95	11.30	5.01
	Bet v 2	43	3	7%	0.29	2.83	20.37	8.93
	Ole e 5	43	3	7%	0.89	9.17	14.42	5.57
	Ole e 1	43	2	5%	0.74	0.85	0.97	0.12
	Phl p 3	43	2	5%	4.63	23.85	43.07	19.22
	Phl p 2	43	2	5%	3.21	15.73	28.24	12.51
	Phl p 5b	43	2	5%	4.68	8.55	12.41	3.86
	Phl p 6	43	2	5%	0.17	1.02	1.86	0.84
	Can f 4	43	2	5%	0.41	2.03	3.66	1.63
	Pla a 1	43	2	5%	0.59	9.94	19.29	9.35
	Bet v 1	43	2	5%	0.61	3.28	5.94	2.67
	Bla g 7	43	2	5%	0.31	2.85	5.39	2.54
	Der p 10	43	2	5%	0.12	3.87	7.63	3.76
	Par j 2	43	2	5%	0.85	39.04	77.22	38.18
	Mus m 1	43	2	5%	0.23	0.58	0.93	0.35
	Blo t 1	43	2	5%	0.29	0.43	0.56	0.14
	Can f 3	43	2	5%	0.12	0.12	0.12	0.00
	Can f 1	43	1	2%	3.37	3.37	3.37	0.00
	Blo t 10	43	1	2%	4.65	4.65	4.65	0.00
	Pla l 1	43	1	2%	0.50	0.50	0.50	0.00
	Equ c 1	43	1	2%	0.79	0.79	0.79	0.00
	Phl p 7	43	1	2%	0.39	0.39	0.39	0.00
	Can f 2	43	1	2%	0.15	0.15	0.15	0.00
	Ole e 3	43	1	2%	0.19	0.19	0.19	0.00
	Ole e 8	43	1	2%	0.12	0.12	0.12	0.00
	Cla h 8	43	0	0%	0.00	n.a	0.00	n.a
	Ole e 10	43	0	0%	0.00	n.a	0.00	n.a
	Ole e 6	43	0	0%	0.00	n.a	0.00	n.a
	Bet v 4	43	0	0%	0.00	n.a	0.00	n.a
	Bla g 1	43	0	0%	0.00	n.a	0.00	n.a
	Art v 6	43	0	0%	0.00	n.a	0.00	n.a
	Asp f 1	43	0	0%	0.00	n.a	0.00	n.a
	Asp f 6	43	0	0%	0.00	n.a	0.00	n.a
	Bla g 2	43	0	0%	0.00	n.a	0.00	n.a
	Bla g 5	43	0	0%	0.00	n.a	0.00	n.a
	Blo t 12	43	0	0%	0.00	n.a	0.00	n.a
	Blo t 8	43	0	0%	0.00	n.a	0.00	n.a
Food	Pen m 2	43	3	7%	0.39	0.70	1.77	0.59
	Ani s 3	43	2	5%	0.17	1.15	2.13	0.98
	Cor a 1.0401	43	2	5%	0.15	0.19	0.23	0.04
	Ara h 1	43	2	5%	0.12	0.12	0.12	0.00
	Ara h 9	43	1	2%	0.12	0.12	0.12	0.00
	Hel as 1	43	1	2%	2.86	2.86	2.86	0.00
	Pen m 1	43	1	2%	5.85	5.85	5.85	0.00
	Pru p 3	43	1	2%	0.12	0.12	0.12	0.00
	Gal d 1	43	1	2%	0.14	0.14	0.14	0.00
	Bos d LF	43	1	2%	0.12	0.12	0.12	0.00
	Ara h 2	43	1	2%	0.12	0.12	0.12	0.00
	Ara h 6	43	0	0%	0.00	n.a	0.00	n.a
	Gly m 4	43	0	0%	0.00	n.a	0.00	n.a
	Ara h 8	43	0	0%	0.00	n.a	0.00	n.a
	Gal d 2	43	0	0%	0.00	n.a	0.00	n.a
	Gal d 3	43	0	0%	0.00	n.a	0.00	n.a
	Gal d 4	43	0	0%	0.00	n.a	0.00	n.a
	Gly m 5	43	0	0%	0.00	n.a	0.00	n.a
	Mal d 1	43	0	0%	0.00	n.a	0.00	n.a
	Pru p 1	43	0	0%	0.00	n.a	0.00	n.a
	Bos d 4	43	0	0%	0.00	n.a	0.00	n.a
	Ara h 3	43	0	0%	0.00	n.a	0.00	n.a
	Bos d 5	43	0	0%	0.00	n.a	0.00	n.a
	Bos d 8	43	0	0%	0.00	n.a	0.00	n.a
	Gad c 1	43	0	0%	0.00	n.a	0.00	n.a
	Gly m 6	43	0	0%	0.00	n.a	0.00	n.a
	Tri a 20	43	0	0%	0.00	n.a	0.00	n.a
	Tri a 37	43	0	0%	0.00	n.a	0.00	n.a
Other	HRP	43	8	19%	0.12	0.26	1.34	0.40
	Ves v 5	43	5	12%	0.12	0.17	0.17	0.02
	Hev b 8	43	2	5%	2.56	9.69	16.82	7.13
	Hev b 5	43	0	0%	0.00	n.a	0.00	n.a
	Api m 2	43	0	0%	0.00	n.a	0.00	n.a
	Ves v 1	43	0	0%	0.00	n.a	0.00	n.a
	Ves v 2	43	0	0%	0.00	n.a	0.00	n.a
	Hev b 3	43	0	0%	0.00	n.a	0.00	n.a
	Api m 1	43	0	0%	0.00	n.a	0.00	n.a

Table S4

Patients from Bahía Blanca			Frequency		IgE (ISU)			
		Total (n)	n	%	Minimum	Median	Maximum	SD
Respiratory	Phl p 1	78	38	49%	0.10	3.56	77.50	15.23
	Fel d 1	78	29	37%	0.10	1.85	47.77	11.99
	Alt a 1	78	27	35%	0.16	13.96	55.51	15.65
	Der p 23	78	26	33%	0.10	5.78	104.14	21.34
	Der p 2	78	26	33%	0.11	8.14	161.42	36.00
	Ole e 1	78	22	28%	0.11	2.55	91.49	19.09
	Der p 1	78	17	22%	0.11	9.74	70.12	17.27
	Phl p 3	78	17	22%	0.33	22.83	86.28	21.96
	Phl p 2	78	16	21%	0.33	4.02	34.88	10.18
	Phl p 5b	78	15	19%	0.15	5.64	194.06	51.07
	Equ c 3	78	12	15%	0.10	0.16	1.62	0.51
	Art v 1	78	12	15%	0.13	2.09	16.25	4.32
	Phl p 6	78	12	15%	0.22	2.24	79.55	21.41
	Blo t 2	78	11	14%	0.10	0.13	0.17	0.02
	Amb a 8	78	11	14%	0.10	0.34	6.47	2.18
	Fel d 2	78	11	14%	0.12	0.24	1.90	0.69
	Der p 7	78	8	10%	0.12	2.26	20.17	7.44
	Der p 21	78	8	10%	0.41	6.02	30.35	11.27
	Can f 4	78	7	9%	0.13	1.87	11.47	4.15
	Can f 1	78	6	8%	0.13	0.54	3.79	1.27
	Der p 5	78	5	6%	1.28	6.90	16.13	5.29
	Der p 37	78	5	6%	0.15	0.64	7.51	2.95
	Ole e 2	78	5	6%	0.10	3.35	7.81	2.55
	Phl p 12	78	5	6%	0.12	5.19	13.87	4.78
	Asp f 3	78	5	6%	0.20	3.11	5.82	2.12
	Pla a 1	78	5	6%	0.11	0.15	12.72	5.02
	Cla h 8	78	5	6%	0.16	0.34	0.45	0.12
	Amb a 4	78	4	5%	0.31	1.18	9.02	3.55
	Bet v 2	78	4	5%	1.71	3.42	7.29	2.05
	Bet v 1	78	4	5%	0.61	2.39	4.56	1.57
	Bla g 7	78	4	5%	0.38	7.66	122.13	50.95
	Der p 10	78	4	5%	0.49	8.05	135.77	56.69
	Der p 4	78	3	4%	0.10	0.12	0.23	0.06
	Amb a 1	78	3	4%	0.13	0.20	22.30	10.43
	Blo t 13	78	3	4%	0.10	0.20	0.38	0.11
	Par j 2	78	3	4%	0.15	0.29	7.21	3.30
	Blo t 10	78	3	4%	0.54	3.73	53.60	24.30
	Pla l 1	78	3	4%	0.10	0.11	0.39	0.13
	Blo t 5	78	2	3%	0.66	1.13	1.59	0.46
	Ole e 5	78	2	3%	0.22	0.37	0.52	0.15
	Equ c 1	78	2	3%	0.20	0.23	0.25	0.02
	Ole e 10	78	2	3%	0.13	0.89	1.66	0.77
	Ole e 6	78	2	3%	0.17	0.18	0.19	0.01
	Blo t 21	78	1	1%	1.01	1.01	1.01	0.00
	Fel d 3	78	1	1%	0.14	0.14	0.14	0.00
	Fel d 4	78	1	1%	11.46	11.46	11.46	0.00
	Mus m 1	78	1	1%	0.91	0.91	0.91	0.00
	Phl p 7	78	1	1%	49.66	49.66	49.66	0.00
	Can f 2	78	1	1%	0.82	0.82	0.82	0.00
	Ole e 3	78	1	1%	58.31	58.31	58.31	0.00
	Bet v 4	78	1	1%	55.10	55.10	55.10	0.00
	Bla g 1	78	1	1%	0.13	0.13	0.13	0.00
	Der p 18	78	0	0%	0.00	n.a	0.00	n.a
	Blo t 1	78	0	0%	0.00	n.a	0.00	n.a
	Can f 3	78	0	0%	0.00	n.a	0.00	n.a
	Ole e 8	78	0	0%	0.00	n.a	0.00	n.a
	Art v 6	78	0	0%	0.00	n.a	0.00	n.a
	Asp f 1	78	0	0%	0.00	n.a	0.00	n.a
	Asp f 6	78	0	0%	0.00	n.a	0.00	n.a
	Bla g 2	78	0	0%	0.00	n.a	0.00	n.a
	Bla g 5	78	0	0%	0.00	n.a	0.00	n.a
	Blo t 12	78	0	0%	0.00	n.a	0.00	n.a
	Blo t 8	78	0	0%	0.00	n.a	0.00	n.a
Food	Ani s 3	78	6	8%	0.10	0.37	39.10	14.25
	Ara h 9	78	5	6%	0.12	0.57	1.45	0.54
	Hel as 1	78	5	6%	0.13	0.83	46.41	18.35
	Pen m 1	78	4	5%	0.64	2.46	91.06	38.64
	Pru p 3	78	4	5%	0.10	0.53	1.01	0.32
	Gal d 1	78	2	3%	0.13	1.37	2.61	1.24
	Ara h 6	78	2	3%	0.22	0.27	0.33	0.06
	Gly m 4	78	2	3%	0.16	0.26	0.36	0.10
	Pen m 2	78	1	1%	0.13	0.13	0.13	0.00
	Cor a 1.0401	78	1	1%	0.66	0.66	0.66	0.00
	Ara h 1	78	1	1%	0.12	0.12	0.12	0.00
	Bos d LF	78	1	1%	0.11	0.11	0.11	0.00
	Ara h 8	78	1	1%	3.00	3.00	3.00	0.00
	Gal d 2	78	1	1%	1.40	1.40	1.40	0.00
	Gal d 3	78	1	1%	1.79	1.79	1.79	0.00
	Gal d 4	78	1	1%	1.00	1.00	1.00	0.00
	Gly m 5	78	1	1%	0.84	0.84	0.84	0.00
	Mal d 1	78	1	1%	2.05	2.05	2.05	0.00
	Pru p 1	78	1	1%	8.49	8.49	8.49	0.00
	Bos d 4	78	1	1%	0.26	0.26	0.26	0.00
	Ara h 2	78	0	0%	0.00	n.a	0.00	n.a
	Ara h 3	78	0	0%	0.00	n.a	0.00	n.a
	Bos d 5	78	0	0%	0.00	n.a	0.00	n.a
	Bos d 8	78	0	0%	0.00	n.a	0.00	n.a
	Gad c 1	78	0	0%	0.00	n.a	0.00	n.a
	Gly m 6	78	0	0%	0.00	n.a	0.00	n.a
	Tri a 20	78	0	0%	0.00	n.a	0.00	n.a
	Tri a 37	78	0	0%	0.00	n.a	0.00	n.a
Other	HRP	78	14	18%	0.10	0.17	0.49	0.12
	Ves v 5	78	7	9%	0.14	0.39	0.75	0.23
	Hev b 8	78	4	5%	1.72	4.07	8.84	2.60
	Hev b 5	78	1	1%	1.39	1.39	1.39	0.00
	Api m 2	78	1	1%	0.30	0.30	0.30	0.00
	Ves v 1	78	0	0%	0.00	n.a	0.00	n.a
	Ves v 2	78	0	0%	0.00	n.a	0.00	n.a
	Hev b 3	78	0	0%	0.00	n.a	0.00	n.a
	Api m 1	78	0	0%	0.00	n.a	0.00	n.a
