# Supplementary material for: Profound Impact of Local Climatic Conditions on IgE Sensitization Profiles: Evidence from Argentine Cities
Source: Int J Mol Sci. 2025 Dec 16;26(24):12101. doi: 10.3390/ijms262412101 (PMC12733070; doi:10.3390/ijms262412101)
Supplement: Supplementary file 1 [file ijms-26-12101-s001.zip › Table S5.pdf]

		Season	Summer			Autumn			Winter			Spring			Annual average/total number of grains or spores
		Months	December	January	February	March	April	May	June	July	August	September	October	November	
Climatological statistics 1991-2020 (SMN Argentina)	La Plata	Average temperature (°C)	21.6	23.1	22.2	20.2	16.4	13	10.1	9.2	11	12.8	15.9	18.8	16.2
		Temperature maximum (°C)	27.5	28.9	27.8	25.8	22.1	18.2	15.1	14.2	16.4	18.1	21	24.4	21.6
		Temperature minimum (°C)	15.8	17.4	16.9	15.2	11.7	8.7	6	5.2	6.3	7.9	10.7	13.4	11.3
		Relative humidity (%)	70.8	72.2	75.7	79.1	82.2	85.1	84.4	83.7	81.4	79	78.6	74.3	78.9
		Precipitation (mm)	93.7	111	112.8	106.5	98.2	78	58.6	78.7	67.5	71.4	101	95.3	1072.7
	Bahia Blanca	Average temperature (°C)	22	23.6	22.1	19.6	15.1	11.4	8.4	7.6	9.6	11.8	15	18.7	15.4
		Temperature maximum (°C)	29.7	31.2	29.6	27	22	17.7	14.5	13.9	16.5	18.9	22.1	26	22.4
		Temperature minimum (°C)	14.5	16.3	15.2	13.4	9.4	6.4	3.5	2.4	4.1	5.6	8.5	11.6	9.2
		Relative humidity (%)	52	53	59.4	65.3	69.9	75.2	73.4	72.3	67	65.2	64.9	58.1	64.6
		Precipitation (mm)	64.7	66.2	69.4	70.6	53.2	41.5	32.6	32.4	31.7	45	69.5	62.3	639.1
Main pollen annual distribution in Bahia Blanca 2005 (grains/m^3)		*Cupressaceae*	+	+	+	+		+	+	+	Peak (8013)	+	+	+	14482.4
		Fraxinus							+	+	+	Peak (4600)	+		5068.6
		Myrtaceae	Peak (2423)	+	+	+							+	+	4952.7
		Poaceae	+	+	+	+	+	+	+	+	+	+	+	Peak (1706)	4289.2
		Amaranthus	+	+	+	Peak (551)	+	+			+	+	+	+	2102.0
		Olea	+										Peak (324)	+	520.0
Anamorphic fungal spore concentration in La Plata (*Alternaria, Cladosporium* etc.)			11000	13500	14500	8500	9500	9000	5000	5000	10000	2500	5500	1000	95000.0
