# Supplementary material for: Profound Impact of Local Climatic Conditions on IgE Sensitization Profiles: Evidence from Argentine Cities
Source: Int J Mol Sci. 2025 Dec 16;26(24):12101. doi: 10.3390/ijms262412101 (PMC12733070; doi:10.3390/ijms262412101)
Supplement: Supplementary file 1 [file ijms-26-12101-s001.zip › Table S6.pdf]

| Rhinoconjunctivits |
|---|
| Have you ever had rhinits?<br>Have you had sneezing, runny, or blocked nose not associated to cold or flu in the past year?<br>Have you had itchy-watery eyes and a stuffy nose in the past year? |
| **Allergic asthma** |
| Have you ever had wheezing or whistling in your chest in the past<br>Have you seen a doctor or been to the ER for wheezing in the past<br>Have you had wheezing in the past year that was so severe you could only speak one or two words at a time between breaths? |
| **Atopic dermatitis** |
| Have you ever had an itchy rash that was coming and going for few<br>Have you had an itchy rash in the past year?<br>Has this itchy rash affected any of the following places: the fold of the elbows, behind the knees, in front of the ankles, under the buttocks, or around the neck, ears, or eyes? |
