# Supplementary figures and images for: Profound Impact of Local Climatic Conditions on IgE Sensitization Profiles: Evidence from Argentine Cities
Source: Int J Mol Sci. 2025 Dec 16;26(24):12101. doi: 10.3390/ijms262412101 (PMC12733070; doi:10.3390/ijms262412101)

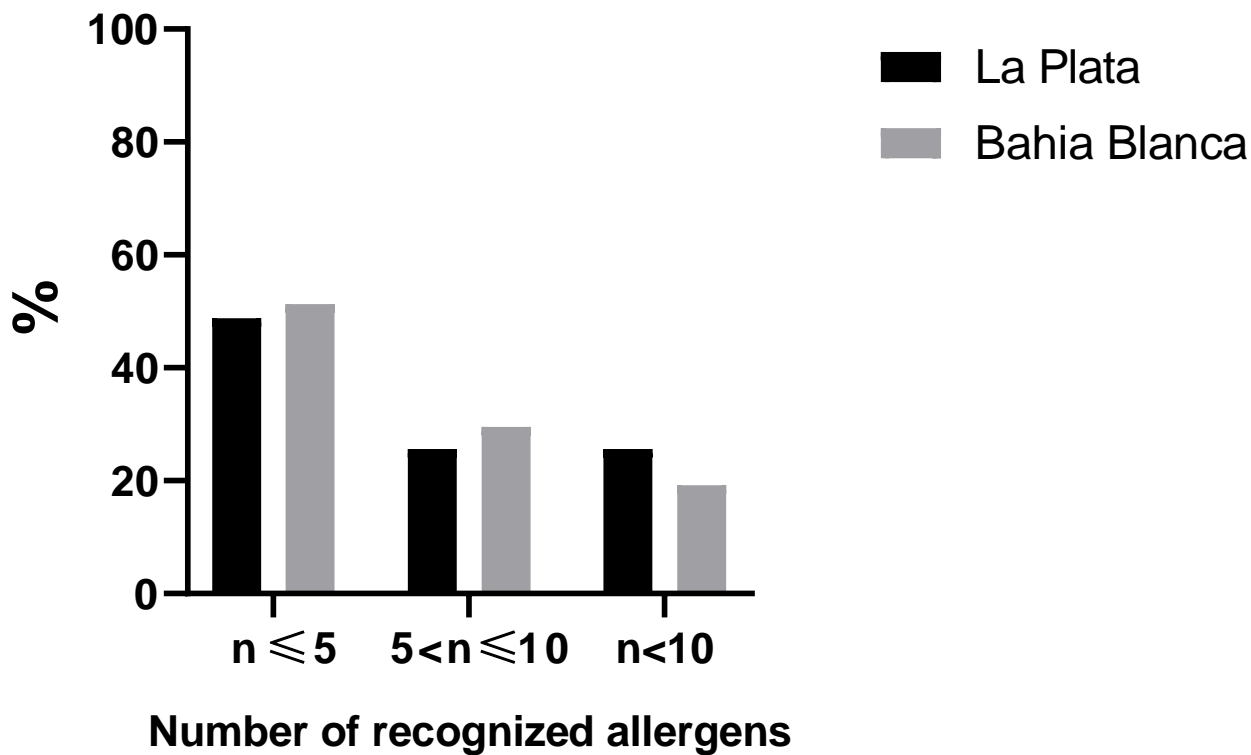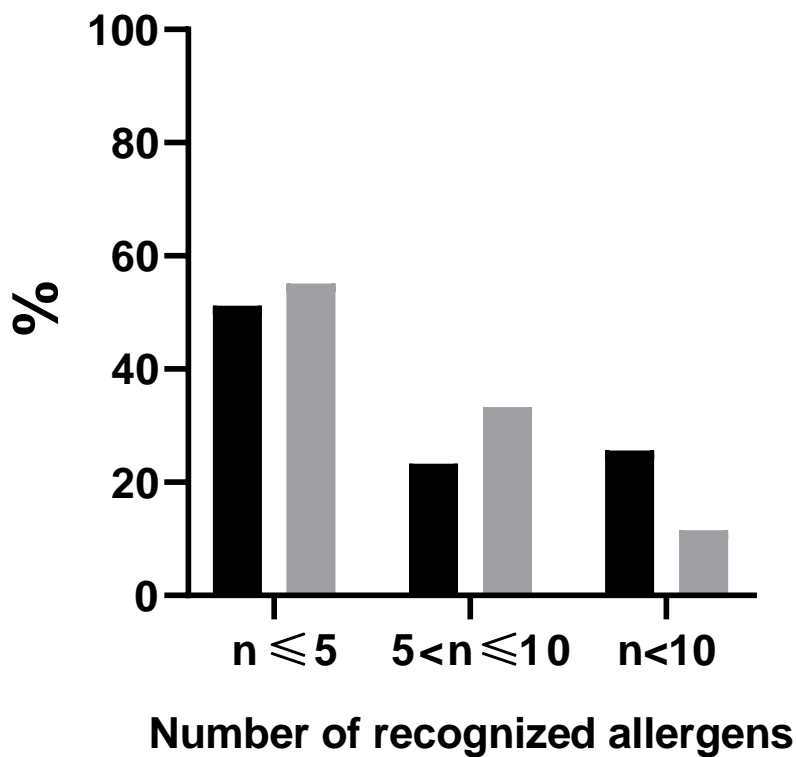

Supplement: Supplementary file 1 [file ijms-26-12101-s001.zip › Figure S1.pdf]
